# Tailoring Thermal Conductivity Anisotropy in Poly(vinylidene fluoride)/Boron Nitride Nanosheet Composites via Processing-Induced Filler Orientation

**DOI:** 10.3390/polym18020291

**Published:** 2026-01-21

**Authors:** Yan-Zhou Lei, De-Xiang Sun

**Affiliations:** 1 Analytical and Testing Center, Southwest Jiaotong University, Chengdu 610031, China; 2 School of Chemistry, Southwest Jiaotong University, Chengdu 610031, China

**Keywords:** PVDF/BNNs composites, processing methods, injection molding, compression molding, thermal conductivity, microstructure

## Abstract

To address the thermal management challenges in electronic devices, this study systematically investigates the effects of injection molding and compression molding on the microstructure and thermal conductivity of poly(vinylidene fluoride)/boron nitride nanosheet (PVDF/BNNs) composites. Using 10 μm diameter BNNs as thermal conductive fillers and PVDF as the matrix, the composites were characterized via scanning electron microscopy (SEM), thermal conductivity measurements, rheological analysis, X-ray diffraction (XRD), and mechanical tests. The results demonstrate that the strong shear stress in injection molding induces significant alignment of BNNs along the flow direction, leading to remarkable thermal conductivity anisotropy. At a PVDF/BNNs mass ratio of 90/10, the in-plane thermal conductivity of the injection-molded composite reaches 1.26 W/(m·K), while the through-plane conductivity is only 0.40 W/(m·K). In contrast, compression molding, which involves minimal shear, results in randomly dispersed BNNs and isotropic thermal conductivity, with both in-plane and through-plane values around 0.41 W/(m·K) at the same filler loading. Both processing methods preserve the coexistence of α- and β-crystalline phases in PVDF. However, injection molding enhances matrix crystallinity through stress-induced crystallization, yielding composites with higher density and superior tensile properties. Compression molding, due to slower cooling, leads to incomplete PVDF crystallization, as evidenced by a shoulder peak near 164 °C in differential scanning calorimetry (DSC) curves. This study elucidates the mechanism by which processing methods regulate the structure and properties of PVDF/BNNs composites, offering theoretical and practical guidance for designing high-performance thermally conductive materials.

## 1. Introduction

With the rapid development of electronic devices toward miniaturization, integration, and high power density, efficient thermal management has become a critical bottleneck restricting their performance improvement and service life extension. Polymer-based thermal conductive composites, combining the excellent processability, lightweight, and corrosion resistance of polymers with the high thermal conductivity of inorganic fillers, have emerged as promising candidates for thermal management materials in various fields such as 5G communications, new energy batteries, and electronic packaging [1,2,3]. However, the final thermal conductivity of these composites is not only determined by the intrinsic properties of the polymer matrix and thermal conductive fillers (e.g., filler thermal conductivity, aspect ratio, and volume fraction) [4,5,6] but also heavily dependent on the processing methods employed during preparation [7,8,9]. Processing methods not only affect the dispersion uniformity of polymer matrices and fillers—directly determining whether a continuous thermal conduction network can be formed [10,11,12]—but also determine the number, type, and distribution of internal defects (such as voids, interfaces, and cracks) in the materials [13,14,15]. These microstructural characteristics collectively influence the heat transfer efficiency within the composites, thereby governing the final thermal conductivity performance [16,17,18].

In the field of polymer-based composite preparation, the selection of an appropriate processing method has long been regarded as a core link in ensuring the target thermal conductivity and comprehensive performance [19,20,21]. Different processing techniques differ significantly in terms of applied force fields (shear and pressure), temperature fields, and material flow behaviors during molding, which, in turn, exert profound impacts on the dispersion state of the polymer matrix and fillers, as well as the quantity and distribution of internal defects [22,23,24]. For instance, uneven filler dispersion may lead to the formation of “thermal insulation regions” in the composite, while excessive internal voids can scatter heat carriers (phonons) and drastically reduce thermal conductivity [25,26,27]. Therefore, a deep understanding of how different molding methods regulate the microstructure (including filler dispersion, orientation, and defect distribution) and macroscopic thermal conductivity of composites is of great significance for optimizing material formulation design, improving processing, and enhancing the application performance of thermal management materials [28,29,30].

Among the numerous processing technologies for polymer composites, compression molding and injection molding are two of the most widely used methods, each with distinct characteristics and application scenarios. Compression molding, a traditional and mature hot-pressing technique, operates by placing the pre-mixed composite materials (such as PVDF/BNNs powder or pellets) into a preheated mold, then applying a certain pressure and maintaining it for a specific period to realize uniform densification of the materials. A key advantage of this molding method is that it avoids the introduction of strong shear stress during the forming process, which helps maintain the original distribution state of the polymer matrix and fillers. This minimizes the orientation of polymer molecular chains and fillers caused by shear flow, thereby yielding composites with relatively isotropic thermal conductivity. Typically, compression molding involves sequential steps such as mold preheating, material loading, pressurization, temperature holding, cooling, and demolding. By precisely controlling parameters including heating rate, molding temperature (generally ranging from the melting point of the polymer to its decomposition temperature), pressure magnitude (usually 10–50 MPa), and holding time, it is possible to prepare composites with uniform microstructure, low void content, and excellent isotropic thermal conductivity. This method is particularly suitable for preparing sheet-like or simple-shaped composite products that require high uniformity in thermal performance.

Injection molding, conversely, is a high-efficiency molding technology that relies on the screw of the injection machine to convey, melt, and mix the composite materials, then inject the molten material into a closed mold cavity at high speed and pressure for rapid cooling and solidification. During this process, the molten composite experiences strong shear stress generated by the screw rotation and the flow through the mold gate and cavity, which may induce significant orientation of polymer molecular chains and anisotropic fillers (such as boron nitride nanosheets). Under specific processing conditions (e.g., appropriate shear rate, mold temperature, and injection speed), this orientation effect can promote the alignment of thermal conductive fillers along the flow direction, thereby forming efficient directional thermal conduction paths in the material and achieving directional enhancement of thermal conductivity. Additionally, injection molding boasts notable advantages such as high production efficiency, short molding cycles (usually ranging from tens of seconds to several minutes), and high product dimensional accuracy, making it highly suitable for mass production of composite products with complex shapes, intricate structures, and strict dimensional requirements—such as heat dissipation components for electronic devices with customized geometries.

In this study, boron nitride nanosheets (BNNs) with a diameter of 10 μm were specifically selected as the thermally conductive filler, since this size enables the composites to achieve an optimal balance between thermal conductivity and mechanical properties [31,32]. BNNs are considered ideal candidates for thermal management fillers due to their ultra-high in-plane thermal conductivity (up to 400 W·m^−1^·K^−1^), excellent electrical insulation, and good chemical stability, which can effectively avoid the problem of electrical short-circuiting in electronic applications. Polyvinylidene fluoride (PVDF) was chosen as the polymer matrix owing to its outstanding mechanical properties, chemical resistance, and good compatibility with inorganic fillers. The research focused on systematically investigating the effects of two typical processing methods—compression molding and injection molding—on the microstructure evolution and thermal conductivity behavior of PVDF/BNNs composites. A series of characterization techniques were employed, including scanning electron microscopy (SEM) to observe the filler dispersion and orientation and X-ray diffraction (XRD) to analyze the crystal structure of the PVDF matrix. Through comparative analysis of the microstructure and thermal conductivity of composites prepared under different molding methods, it was found that composites fabricated by compression molding exhibit more uniform filler dispersion without obvious orientation, accompanied by lower internal void content, thus demonstrating better isotropic thermal conductivity. In this study, we systematically investigated and compared the orientation behavior of 10 μm hexagonal boron nitride (BN) platelets in polyvinylidene fluoride (PVDF) composites prepared by two processes: compression molding and injection molding. We also clarified the intrinsic correlation between processing method, filler orientation degree, and thermal conductivity of the composites. The innovations of this study are mainly reflected in two aspects: first, scanning electron microscopy (SEM) was used to systematically characterize the orientation morphology of BN platelets under different processing conditions. Combined with image analysis technology, the differences in the orientation degree of BN were quantitatively evaluated, and the regulation law of processing conditions on the filler orientation state and its correlation with thermal conductivity were clarified. Second, the mechanism by which processing-induced orientation regulates the anisotropic thermal conductivity of PVDF/BN composites was revealed, providing a theoretical basis for the controllable preparation of composites with specific thermal properties.

The expected outcome of this study is to propose a feasible strategy that can balance the thermal conductivity and processing efficiency of PVDF/BN composites. This strategy can provide guidance for the design and fabrication of high-performance thermal management materials in practical engineering applications. Specifically, the optimized composites are anticipated to serve as reliable heat-dissipating substrates for high-power electronic devices such as lithium-ion battery packs and microprocessors. Moreover, the proposed method is scalable for industrial production, thus facilitating the widespread adoption of PVDF/BN composites in advanced thermal management systems of electric vehicles and aerospace equipment.

## 2. Experimental Section

### 2.1. Experimental Raw Materials

**Figure 1:**
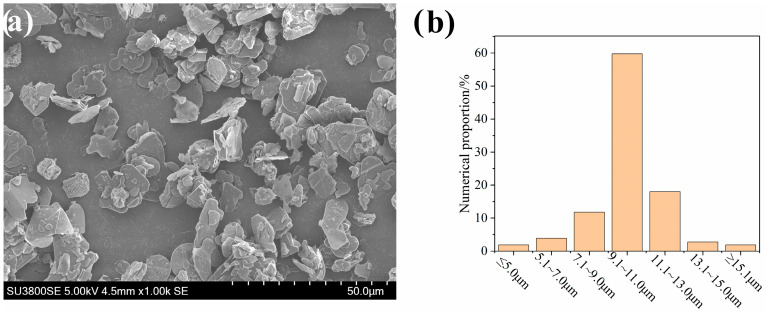
(**a**) SEM morphology of boron nitride; (**b**) particle size statistics of boron nitride.

Boron nitride (~10 μm) was produced by Liaoning Pengda Technology Co., Ltd., Yingkou, China (the morphology and particle size of boron nitride were shown in Figure 1), and polyvinylidene fluoride (FR903) was produced by Shanghai Huayi Sanaifu New Material Co., Ltd. (Shanghai, China).

### 2.2. Sample Preparation Methods

Boron nitride (BN) was exfoliated using a planetary ball mill to prepare exfoliated boron nitride (BNNs). The specific process was as follows: the ball-to-material mass ratio was 100/1, distilled water was used as the dispersant, and ball milling was conducted at 600 rpm for 48 h, followed by freeze-drying and sieving [33].

PVDF and BNNs (~10 μm) were weighed at mass ratios of 90/10, 80/20, 70/30, and 60/40, respectively, and the mixtures were melt-blended in a torque rheometer at 210 °C and 30 rpm for 6 min; after cooling to room temperature and cryogenic crushing, the resultant pellets were split into two portions, with one portion hot-pressed at 10 MPa and 200 °C for 10 min followed by cold-pressing at 8 MPa for another 10 min to obtain bulk PVDF/BNNs composites and the other portion fabricated into target specimens via injection molding under the parameters of 600 bar injection pressure, 190 °C barrel temperature, 25 °C mold temperature, and 30 s cooling time. The molds for both compression molding and injection molding were designed for thermal conductivity testing samples, circular discs with a diameter of 30 mm and thickness of 4 mm.

### 2.3. Characterization Methods

#### 2.3.1. Scanning Electron Microscope (SEM) Analysis

Samples were fully immersed in liquid nitrogen for 5 min, and cross-sectional samples were obtained by quenching and breaking. All samples were subjected to gold ion sputtering treatment with a thickness of 10 nm before testing. Microscopic characterization was performed using a JEOL JSM 7800F (JEOL Ltd., Tokyo, Japan) field emission scanning electron microscope at an acceleration voltage of 2.7 kV and a working distance of 9.3 mm.

#### 2.3.2. Thermal Conductivity Test

Thermal conductivity was tested using a TPS 2500 S thermal conductivity meter (Hot Disk AB, Gothenburg, Sweden) from Hot Disk. The instrument was preheated for 30 min before testing. Samples were circular discs with a diameter of 30 mm and thickness of 4 mm. Each sample was tested 5 times, and the average value was taken. The testing environment was 20 ± 3 °C, with a humidity of 40–60%.

#### 2.3.3. Rheological Test

Dynamic frequency scanning was performed using an ARES-G2 rotational rheometer (TA Instruments, New Castle, DE, USA). The scanning range was 0.1–100 Hz, the test temperature was 200 °C, a φ25 mm parallel plate fixture was used, and the fixed strain was 1%. The plate gap was set at 1 mm.

#### 2.3.4. X-Ray Diffraction (XRD) Test

Crystal structure characterization was conducted using an Empyrean X-ray diffractometer (Malvern Panalytical Ltd., Malvern, UK). The scanning angle range was 10~80°, and the scanning step was 10°/min.

#### 2.3.5. Fourier Transform Infrared Spectroscopy (FTIR) Test

Attenuated total reflection–Fourier transform infrared spectroscopy (ATR-FTIR) was measured using an iS 50 infrared spectrometer (Thermo Fisher Scientific Inc., Waltham, MA, USA). The number of scans was 32, the resolution was 4 cm^−1^, and the acquisition wavenumber range was 4000–400 cm^−1^. The testing environment was 20 ± 3 °C, with a humidity of 40–60%.

#### 2.3.6. Differential Scanning Calorimetry (DSC) Test

DSC testing was performed using a DSC 2500 from TA Instruments. Samples (3~5 mg) were heated from 30 °C to 230 °C at a rate of 10 °C/min, and the heat flow curve was recorded. Nitrogen was purged at a flow rate of 50 mL/min during the DSC test.

#### 2.3.7. Tensile Strength Test

Tensile testing was conducted using an RGM-6005 universal testing machine (Reger Instruments Co., Ltd., Chengdu, China) according to the standard. Samples were dumbbell-shaped, with a cross-sectional area of 5 × 1 mm. A stress of 2 kN was applied, and the tensile rate was 50 mm/min. Each sample was tested 5 times, and the average value was taken. The testing environment was 20 ± 3 °C, with a humidity of 40–60%.

#### 2.3.8. Density Test

True density was measured using an AccuPyc Ⅱ 1340 true density meter (Micromeritics Instrument Corp., Norcross, GA, USA). Before testing, calibration was performed using a φ10 mm standard steel ball. A 10 cm^3^ measuring cell was used, and the sample volume exceeded half of the cell. Helium was used during testing, and the environmental temperature was 20 ± 3 °C, with a humidity of 40–60%.

## 3. Results and Discussion

### 3.1. Microscopic Morphology

**Figure 2:**
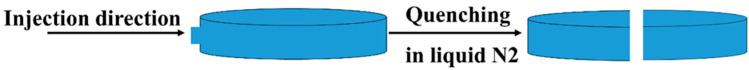
Schematic diagram of the quenching direction of the injection-molded sample.

The shear stress direction in injection molding was the in-plane direction of the disc, and the mold gate position is shown in Figure 2. Samples were immersed in liquid nitrogen for 5 min, then quickly quenched and broken to obtain composite cross-sections, which were sputter-coated with gold and observed under a scanning electron microscope at 2.70 kV.

**Figure 3:**
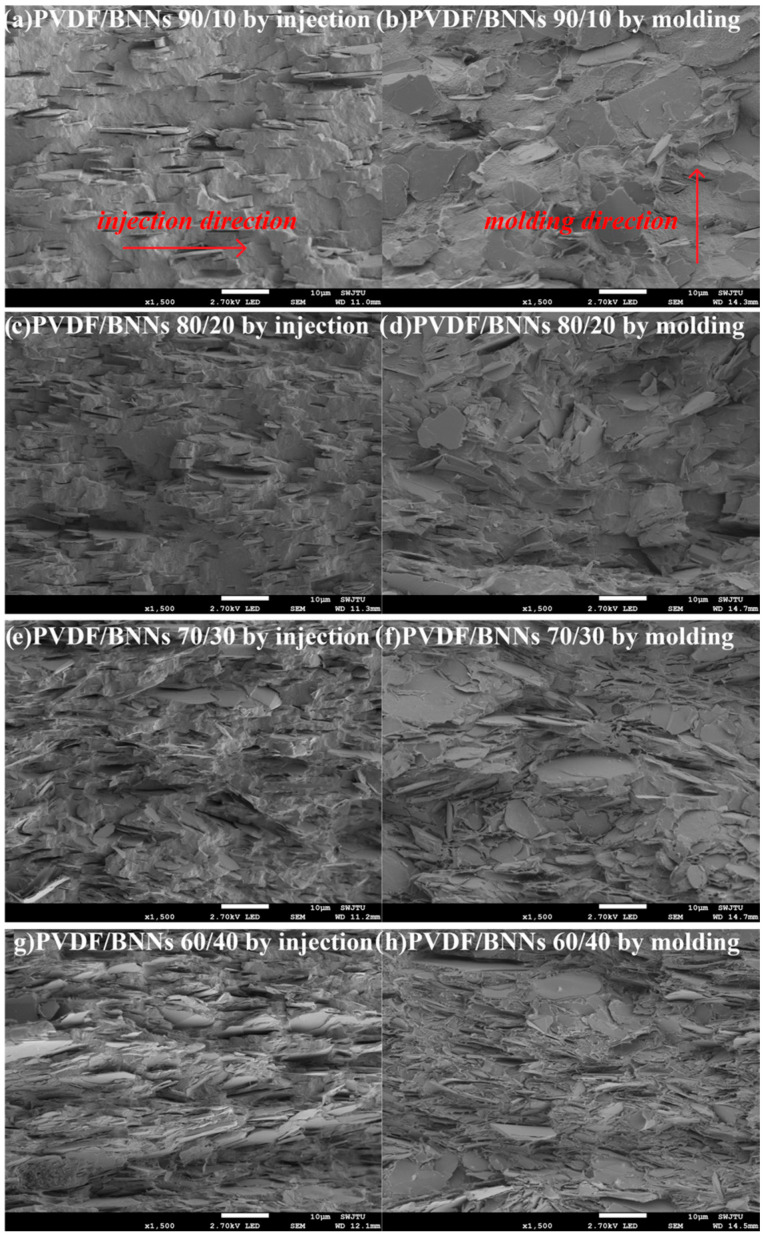
SEM images of the quenched cross-sections of PVDF/BNNs composites prepared by injection molding and compression molding.

As shown in the SEM images of the quenched cross-sections of PVDF/BNNs composites, BNNs were uniformly dispersed in the matrix without agglomeration in both processing methods. When the mass ratio of PVDF/BNNs was 90/10, the amount of BNNs in composites prepared by both molding methods was too small to form a complete thermal conduction network. However, during the injection molding filling process, the PVDF/BNNs melt was pushed by the piston pressure through the nozzle into the mold, causing BNNs in the melt to highly orient along the shear stress direction, followed by rapid cooling in the mold. Thus, BNNs in the obtained sample exhibit significant anisotropy—highly oriented along the injection direction, i.e., the in-plane direction of the disc. As shown in Figure 3a,d,e,g, BNNs, as two-dimensional fillers, were highly oriented along the injection stress direction (i.e., parallel to their in-plane direction). In Figure 3a, although the number of BNNs was insufficient to construct a thermal conduction path, BNNs were connected end to end along the shear stress direction, forming a two-dimensional thermal conduction path in the arrow direction. In contrast, composites prepared by compression molding with the same PVDF/BNNs mass ratio show random arrangement and no orientation of BNNs in the matrix due to the absence of stress during processing, thus failing to form a thermal conduction path.

With the further increase in BNNs proportion, when the PVDF/BNNs mass ratio reaches 80/20, BNNs in composites prepared by compression molding were sufficiently numerous to overlap and establish a thermal conduction network. However, due to the poor binding force between BNNs and the PVDF matrix and the influence of BNNs on the free movement ability of PVDF molecular chains, the increase in BNNs content also leads to an increase in defects in the composites. As shown in the red frames of Figure 3e,f,g, void defects easily form near BNNs, which affect thermal conductivity, mechanical properties, etc.

### 3.2. Thermal Conductivity Performance

**Figure 4:**
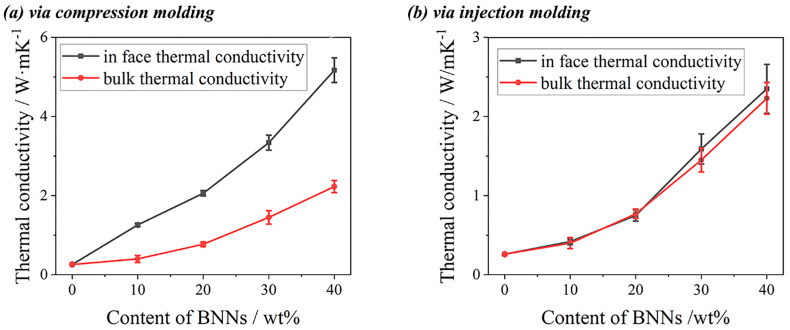
In-plane and bulk thermal conductivities of PVDF/BNNs composites: (**a**) compression-molded; (**b**) injection-molded.

The in-plane and bulk thermal conductivities of composites were tested using a TPS 2500 thermal conductivity meter. As shown in Figure 4, large-diameter BNNs significantly improve the thermal conductivity of composites, and the thermal conductivity of composites obtained by both processing methods was substantially enhanced. Combined with Figure 3, due to the strong shear stress during injection molding, BNNs were highly oriented along the injection direction (i.e., in-plane direction). When the PVDF/BNNs mass ratio was 90/10, BNNs in the in-plane direction contacted each other to form a thermal conduction path. In thermal conductivity testing, PVDF/BNNs composites (injection-molded) exhibit significant anisotropy; the in-plane thermal conductivity reaches 1.26 W/(m·K), while the bulk thermal conductivity was only 0.40 W/(m·K). In contrast, samples prepared by compression molding have no shear stress during processing, and BNNs were randomly distributed in the PVDF matrix, showing isotropic properties. Thus, there was no significant difference between in-plane and bulk thermal conductivities. When the PVDF/BNNs mass ratio was 90/10, the in-plane and bulk thermal conductivities were 0.41 W/(m·K) and 0.42 W/(m·K), respectively, close to the bulk thermal conductivity of injection-molded samples.

### 3.3. Rheological Properties

**Figure 5:**
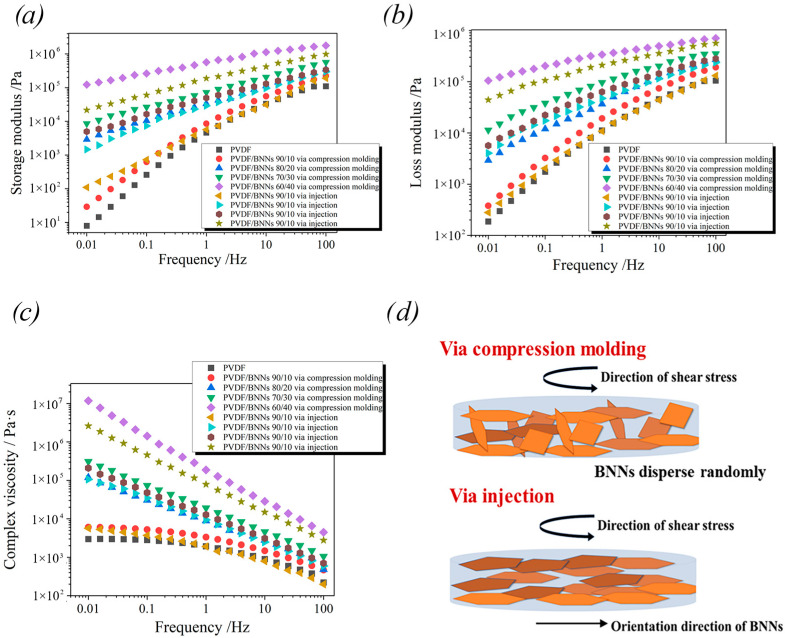
Rheological curves of PVDF/BNNs composites: (**a**) storage modulus, (**b**) loss modulus, (**c**) complex viscosity, and (**d**) schematic diagram of the shear direction.

To further investigate the internal structure of composites, rotational rheology was used to characterize composites prepared by different molding methods. Figure 5a, Figure 5b, and Figure 5c the curves of storage modulus, loss modulus, and complex viscosity with frequency, respectively. In the low-frequency region, pure PVDF and PVDF/BNNs 90/10 composites exhibit low storage modulus. Under long-term shear action (low frequency), molecular chains can respond promptly to shear stress, i.e., undergo corresponding deformation. With the increase in frequency, strain shows certain hysteresis, macroscopically manifested as an increase in storage modulus. As the content of BNNs in the system increases, the restriction on the movement of PVDF chain segments was enhanced, and molecular chains cannot promptly produce corresponding strain under shear stress, resulting in an increase in storage modulus in the low-frequency region. At a frequency of 0.01 Hz, the storage moduli of PVDF/BNNs 90/10 composites prepared by compression molding and injection molding reach 1.2 × 10^5^ Pa and 2.2 × 10^4^ Pa, respectively, which were 5 and 4 orders of magnitude higher than that of pure PVDF. Under the same PVDF/BNNs mass ratio, composites prepared by compression molding exhibit higher storage modulus than those by injection molding. This is because two-dimensional filler BNNs in composites prepared by injection molding were highly oriented, and the orientation direction was parallel to the shear stress direction during rheological testing (as shown in Figure 5d). Under shear stress, the entanglement degree between BNNs and PVDF chain segments was lower, resulting in relatively less restriction on the free movement of chain segments and, hence, lower storage modulus than composites prepared by compression molding.

Similarly, pure PVDF and PVDF/BNNs 90/10 composites show low loss modulus in the low-frequency region, only 10^2^. As the content of BNNs in the system increases, the rheological test was conducted at a high temperature of 200 °C, where PVDF has transitioned from the rubbery state to the molten state. Under shear stress, molecular chains in the system disentangle and start to move freely, while BNNs remain in a solid state in the system, generating significant friction with the sheared molecular chains, leading to an increase in loss modulus. The loss modulus of compression-molded PVDF/BNNs 90/10 composites reaches 1.05 × 10^5^ Pa at 0.01 Hz, which is 3 orders of magnitude higher than that of pure PVDF. Horizontal comparison of the two series of composites shows that composites prepared by compression molding exhibit higher loss modulus at the same PVDF/BNNs mass ratio. As shown in Figure 5d, in composites obtained by injection molding, the shear stress direction was parallel to the direction of two-dimensional fillers, so the PVDF melt between layers avoids friction with BNNs. In compression-molded composites, BNNs were randomly dispersed without orientation, and polymer molecular chains will generate significant friction with BNNs under shear stress, resulting in higher loss modulus.

In Figure 5c, pure PVDF and PVDF/BNNs 90/10 composites exhibit obvious Newtonian platforms, i.e., these three systems show almost no dependence on frequency. This is because BNNs in these systems impose no or little restriction on the free movement of molecular chain segments. Under low-frequency shear stress, the entanglement and disentanglement of molecular chains were almost balanced, resulting in low complex viscosity. With the increase in shear stress frequency, the internal structure of the system was destroyed, and the disentanglement rate of molecular chain segments was faster than the entanglement rate, leading to a decrease in complex viscosity. As the content of BNNs in the system increases, the Newtonian platform gradually disappears. When the PVDF/BNNs mass ratio reaches 80/20 or above, composites all exhibit typical “shear thinning” behavior. As shown in the SEM images in Section 2.1, when the PVDF/BNNs mass ratio reaches 80/20 or above, the number of BNNs was sufficient to form a complete three-dimensional network through end-to-end connection. This three-dimensional network remains in a solid state during rheological testing and frictions with the polymer matrix under shear, severely restricting the free movement of molecular chain segments. In the low-frequency region, the disentanglement rate of molecular chains was faster than the entanglement rate, and, with the increase in frequency, the disentanglement rate of molecular chains was further faster than the entanglement rate, showing more obvious shear thinning. Similarly, the strong shear stress of the piston on the polymer melt during injection molding leads to the highly oriented structure of BNNs in the matrix, weakening the interaction between BNNs and PVDF molecular chains, macroscopically manifested as reduced shear thinning degree.

Summarizing the data of storage modulus, loss modulus, and complex viscosity with the PVDF/BNNs mass ratio, it was found that these three parameters all undergo abrupt changes at the PVDF/BNNs mass ratio of 80/20, which corresponds to the quenched surface morphology and thermal conductivity of the composites. After the PVDF/BNNs mass ratio reaches 80/20, BNNs in the matrix were connected end to end to form a complete thermal conduction path.

### 3.4. Crystallization Properties

**Figure 6:**
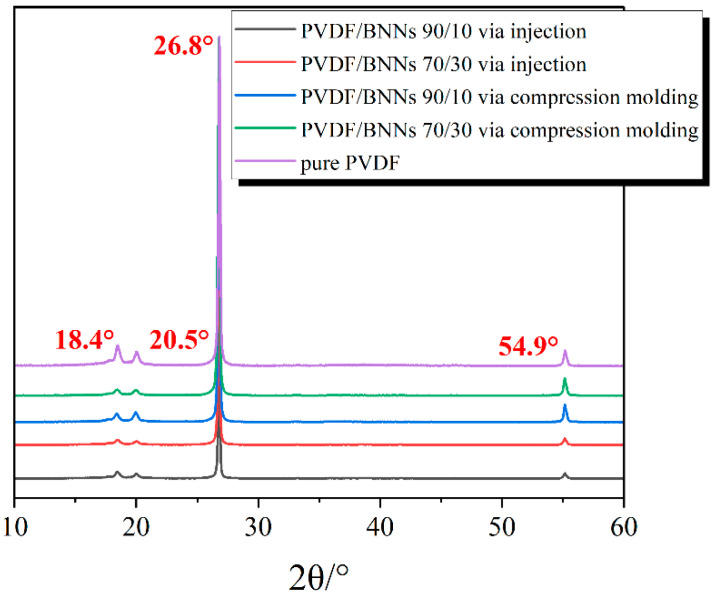
XRD patterns of PVDF/BNNs composites prepared by injection molding and compression molding (From top to bottom, the samples correspond PVDF/BNNS mass ratios of 90/10, 70/30 prepared by injection, PVDF/BNNS mass ratios of 90/10, 70/30 prepared by compression molding, and pure PVDF, respectively).

To investigate the effect of processing methods on the crystal form of PVDF, X-ray diffraction (XRD) was used to characterize the sample structure. The test conditions were scanning from 10° to 60° at a step of 10°/min. Figure 6 shows the XRD spectra of PVDF/BNNs 90/10 and 70/30 composites prepared by different molding methods. The diffraction peaks at 2θ of 18.4° (020) and 26.8° (021) were assigned to the α-crystal form of PVDF, the diffraction peak at 2θ of 20.5° (200) was assigned to the β-crystal form of PVDF, and the diffraction peak at 54.9° corresponds to the (004) crystal plane of BNNs. It can be seen that, regardless of the molding method (compression molding or injection molding) and the PVDF/BNNs mass ratio, the XRD patterns only exhibit the above four diffraction peaks, indicating that the composites coexist with α and β crystal forms [34,35].

**Figure 7:**
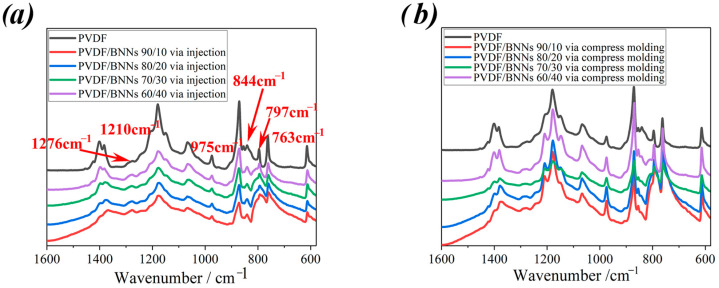
FTIR spectra of PVDF/BNNs composites prepared by different molding methods, (**a**) PVDF/BNNs composites prepared via injection, (**b**) PVDF/BNNs composites prepared via compress molding (From top to bottom, the samples correspond to pure PVDF, PVDF/BNNS mass ratios of 90/10, 80/20, 70/30, and 60/40, respectively).

To further discuss the effect of compression molding and injection molding on the crystal form of PVDF/BNNs composites, the attenuated total reflection–Fourier transform infrared spectroscopy (ATR-FTIR) of all samples was tested, and the data are shown in Figure 7. The characteristic absorption peaks of PVDF α-crystal form were at 763 cm^−1^, 797 cm^−1^, 975 cm^−1^, and 1210 cm^−1^, while those of β-crystal form were at 844 cm^−1^ and 1276 cm^−1^ [36,37]. For different molding methods and PVDF/BNNs mass ratios, the relative intensities of absorption peaks in the FTIR spectra basically do not change, which is consistent with the XRD patterns, i.e., both α and β crystal forms coexist in PVDF/BNNs composites prepared by injection molding and compression molding.

**Figure 8:**
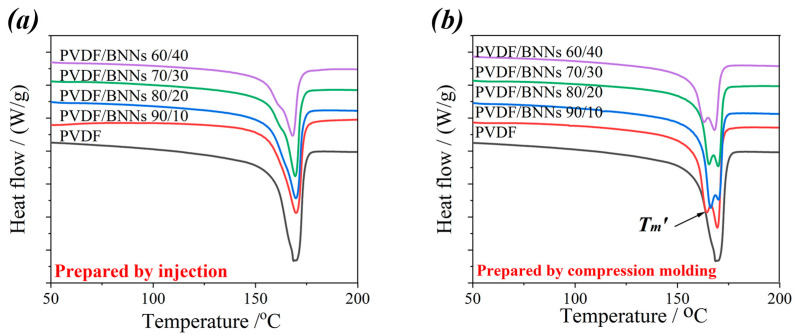
DSC curves of PVDF/BNNs composites prepared by different molding methods (upward exothermic), (**a**) PVDF/BNNs composites prepared via injection, (**b**) PVDF/BNNs composites prepared via compress molding (From top to bottom, the samples correspond to PVDF/BNNS mass ratios of 60/40, 70/30, 80/20, 90/10, and pure PVDF, respectively).

Furthermore, the crystallization properties of the composites were characterized using differential scanning calorimetry (DSC). Since this section focuses on the effect of processing methods on the crystallization properties of the composites, the **t**hermal history of the samples was not eliminated during DSC testing. Samples were heated from 30 °C to 230 °C at a rate of 10 °C/min, and the heat flow curves during heating were recorded. The DSC curves of all samples and selected DSC parameters are presented in Figure 8 and Table 1, respectively.

As shown in Figure 8 and the table, there is no significant difference in the melting temperature (*Tm*) of composites prepared with different processing methods or PVDF/boron nitride nanosheet (BNNs) mass ratios, indicating that the crystal form of PVDF remains unchanged. However, as the BNNs content increases in the PVDF/BNNs mass ratio, the melting enthalpy (Δ*Hm*) of the composites shows an upward trend, meaning the crystallinity increases accordingly. When the PVDF/BNNs mass ratio is 60/40, the melting enthalpies of the composites prepared by injection molding and compression molding reach 54 J/g and 56.6 J/g, respectively—marking a noticeable increase in crystallinity compared to the samples with the lowest BNNs content (90/10: 46.6 J/g for injection molding; 50.4 J/g for compression molding).

Although the introduction of BNNs increases the melt viscosity during processing, BNNs themselves act as heterogeneous nucleation sites for PVDF molecular chains, promoting the regular arrangement of chains in the crystalline region. Consequently, the crystallinity increases with the rise in BNNs content. When comparing composites prepared by different processing methods at the same PVDF/BNNs mass ratio, it is found that the melting enthalpy of compression-molded samples is slightly higher than that of injection-molded samples. This indicates that the static pressure during compression molding is more conducive to the ordered stacking of PVDF chains via BNNs, resulting in higher crystallinity. In addition, a shoulder peak appears at 164–165 °C in the DSC curves of compression-molded samples. Combined with XRD results (no new diffraction peaks, i.e., no new crystal forms are generated), this shoulder peak is attributed to the incomplete crystallization of some PVDF chains, caused by slow cooling and the absence of strong shear stress during compression molding.

### 3.5. Mechanical Properties

**Figure 9:**
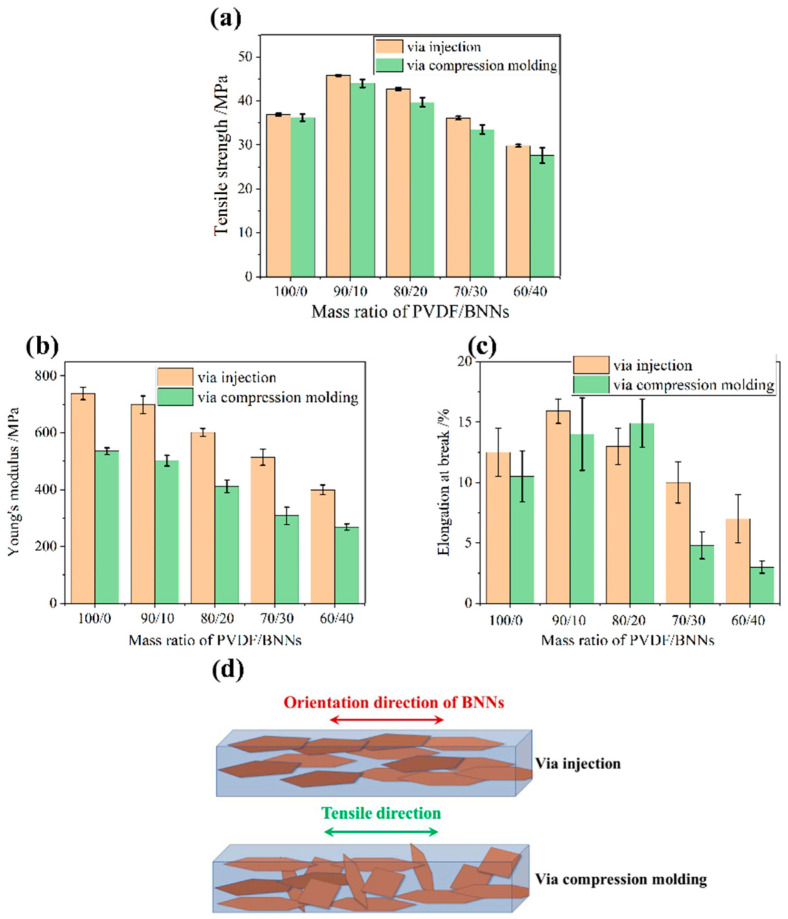
Mechanical properties of PVDF/BNNs composites: (**a**) tensile strength, (**b**) Young’s modulus, (**c**) elongation at break, and (**d**) schematic diagram of the tensile direction.

To discuss the effect of injection molding and compression molding on the mechanical properties of PVDF/BNNs composites, tensile testing was performed on the samples, and the test results are shown in Figure 9. The addition of large-diameter (10 μm) BNNs significantly enhances the properties of PVDF composites, and the tensile strength of most PVDF/BNNs composites was higher than that of pure PVDF. First, consider composites prepared by compression molding: when the BNNs/PVDF mass ratio increases from 0/100 to 10/90, the tensile strength of the composite system reaches the highest value, increasing from 36.2 MPa to 44.0 MPa. This is because BNNs themselves have excellent mechanical strength, leading to good reinforcement of the polymer matrix. However, due to the large polarity difference between ceramic fillers and the polymer matrix, the interfacial bonding force between BNNs and the PVDF matrix was weak. When the material was subjected to stress, the interfacial bonding site was easily damaged, leading to material fracture. Therefore, with the increase in BNNs content, more filler-matrix interfacial bonding sites were generated, resulting in a decrease in tensile strength. When the PVDF/BNNs mass ratio was 60/40, the tensile strength of the composite decreases to 27.6 MPa.

Next, consider composites prepared by injection molding: they exhibit the same change trend as compression-molded PVDF/BNNs composites, first increasing and then decreasing. The tensile strength also reaches the highest value of 45.8 MPa at the PVDF/BNNs mass ratio of 90/10, which was 24.1% higher than that of pure PVDF. With the further increase in BNNs content, the tensile strength of the composite decreases due to the increase in interfaces. When the PVDF/BNNs mass ratio was 60/40, the tensile strength of the composite was only 29.8 MPa.

**Figure 10:**
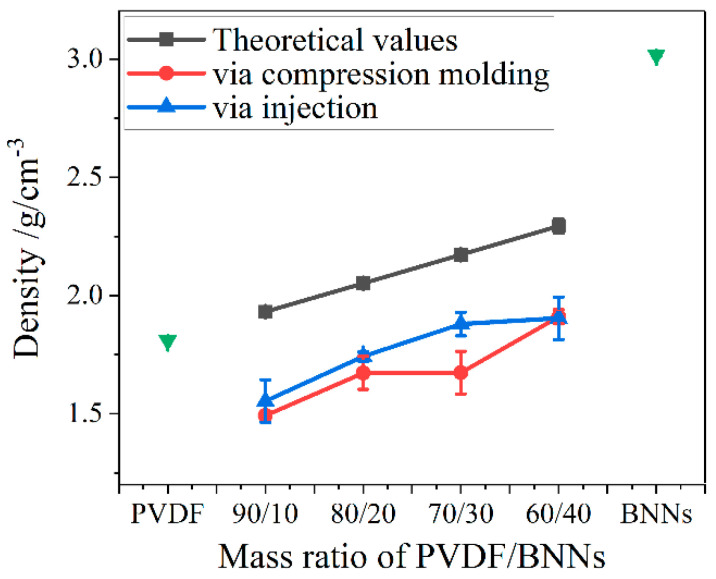
Densities of PVDF, BNNs, and PVDF/BNNs composites.

To better understand the differences in mechanical properties of composites prepared by the two molding methods, true density testing was performed on PVDF, BNNs, and composites using a true density meter. The true density meter uses small-molecule helium gas and determines the true volume of the sample by measuring the change in gas volume after placing the sample in the test chamber based on Boyle’s law (PV = nRT). The true density calculation formula is true density = mass/true volume. This method can eliminate the possibility of sample dissolution in the immersion method, does not damage the sample, and, since the gas can penetrate into the tiny pores of the sample, the measured volume was closer to the true volume of the sample. The densities of each composite are shown in Figure 10. The densities of PVDF and BNNs were 1.8 g/cm^3^ and 3.0 g/cm^3^, respectively.

As shown in Figure 10, the density of composites prepared by both molding methods was lower than the theoretical value due to many defects inside the material. Comparing composites prepared by the two molding methods, it was found that the density of injection-molded composites was higher than that of compression-molded composites. This was because the strong shear stress during injection molding expels part of the bubbles in the material, and stress-induced crystallization promotes the regular and close arrangement of PVDF molecular chains, macroscopically manifested as higher density.

Comparing the variation laws of tensile strength with BNNs content in composites prepared by the two molding methods, although the general trend was first increase and then decrease, at the same PVDF/BNNs mass ratio, injection-molded samples always exhibit more excellent mechanical properties. This was because injection molding leads to the high orientation of BNNs fillers inside the composite, as shown in Figure 9d, where the orientation direction of two-dimensional filler BNNs was the same as the stress direction during tensile testing. First, in the tensile stress direction, the regular B-N covalent bonds in BNNs replace some random PVDF chain segments to bear the tensile stress, leading to an increase in tensile strength. Second, void defects easily occur at the interface between the edges of two-dimensional fillers and the matrix. Since the tensile stress direction was consistent with the filler orientation direction, the “stress concentration” phenomenon during stretching was reduced, also leading to an increase in tensile strength. Third, injection-molded samples have fewer voids and bubble defects. Fourth, the strong shear action of the injection molding piston on the polymer melt has a stress-induced crystallization effect, promoting the regular arrangement of PVDF molecular chain segments and improving the crystallinity of PVDF. In summary, injection molding was more conducive to preparing composites with more excellent tensile properties.

## 4. Conclusions

In summary, this study employed boron nitride nanosheets (BNNs) with a diameter of 10 μm as thermally conductive fillers to systematically investigate the effects of distinct processing techniques on the thermal conductivity of PVDF/BNNs composites. The intense shear force inherent in the injection molding process induces pronounced alignment of BNNs along the shear direction (i.e., the in-plane direction). Owing to their large diameter, BNNs can interconnect and overlap in the in-plane direction even at a relatively low mass fraction, thereby constructing efficient heat transfer pathways. In contrast, compression molding lacks such shear-induced orientation; in the absence of shear stress and with a slower cooling rate, the fillers are randomly dispersed throughout the PVDF matrix, resulting in composites with isotropic thermal conductivity. Specifically, when the mass ratio of PVDF to BNNs was set at 90/10, BNNs in the injection-molded composites formed continuous thermal conduction networks in the in-plane direction, leading to significant thermal anisotropy. Thermal conductivity tests demonstrated that the in-plane thermal conductivity of injection-molded PVDF/BNNs composites reached 1.26 W/(m·K), whereas the through-plane thermal conductivity was merely 0.40 W/(m·K). For compression-molded composites, the absence of stress-induced crystallization and the slow cooling rate give rise to incomplete crystallization of the PVDF matrix, which is evidenced by the presence of a shoulder peak at approximately 164 °C in their thermal analysis profiles. These findings provide a feasible strategy for the customized preparation of PVDF/BNNs composites with tunable thermal conductivity anisotropy, which can be further applied in the design and fabrication of high-performance thermal management devices for electronic packaging and flexible sensors.

## Figures and Tables

**Table 1:** DSC Parameters of PVDF and PVDF/BNNs composites prepared by injection and compression molding.

Sample		T_m_/°C	T_m_^′^/°C	△H_m_/J/g
PVDF		170.0		47.6
Prepared by injection	PVDF/BNNs 90/10	170.0	/	46.6
	PVDF/BNNs 80/20	169.7	/	52.5
	PVDF/BNNs 70/30	169.4	/	54
	PVDF/BNNs 60/40	168.2	/	54
Prepared by compression molding	PVDF/BNNs 90/10	169.4	164.2	50.4
	PVDF/BNNs 80/20	170.1	163.3	50.8
	PVDF/BNNs 70/30	169.9	165.4	53.2
	PVDF/BNNs 60/40	168.1	163.0	56.6

## Data Availability

The original contributions presented in this study are included in the article. Further inquiries can be directed to the corresponding author.
